# Symptomatic malignant spinal cord compression in children: a single-center experience

**DOI:** 10.1186/s13052-019-0671-5

**Published:** 2019-07-12

**Authors:** Lucia De Martino, Piero Spennato, Simona Vetrella, Maria Capasso, Carolina Porfito, Serena Ruotolo, Massimo Eraldo Abate, Giuseppe Cinalli, Lucia Quaglietta

**Affiliations:** 1 Department of Pediatric Oncology, Santobono-Pausilipon Children’s Hospital, Posillipo Street, 226, 80122 Naples, Italy; 2 Department of Pediatric Neurosurgery, Santobono-Pausilipon Children’s Hospital, Naples, Italy

**Keywords:** Spinal cord compression, Extramedullary, Intradural, Intramedullary, Motor deficit, Sphincter dysfunction, Pain

## Abstract

**Background:**

Malignant spinal cord compression (MSCC) is associated withpoor prognosis and may lead to permanent paralysis, sensory loss, and sphincter dysfunction. Very limited data are available on incidence and etiology of MSCC in pediatric population. We aimed to examine etiology, clinical presentation and treatment of pediatric patient with MSCC admitted to the Santobono-Pausilipon Children’s Hospital, Naples, Italy.

**Methods:**

Forty-four children under 18 yearsadmitedsince 2007 and assessed for MSCC clinical presentations, evaluation, and treatment.were retrospectively collected from our institutional pediatric oncology and neurosurgery database.

**Results:**

The median age at time of MSCC diagnosis was 52 months, with a peak in young (≤3 years) patients. The leading cause of MSCC was extramedullary tumors (63.6%), in particular neuroblastoma (27.2%) followed by Ewing sarcomas (15.9%). Cord compression was the presenting feature of a new malignancy in 33 (75%) patients, and a consequence of metastatic disease progression or relapse in the remaining 11 (25%) patients. Motor deficit was the initial symptoms of spinal compression in all patients, while pain was present in about 60% of patients, followed by sphincteric deficit (43.2%). The primary tumor site was located in the neck in 3 (6.8%) patients, thorax in 16 (36.4%), cervico-thoracic region in 3 (6.8%), thoraco-lumbar region in 8 (18.2%), abdomen in 5 (11.4%), lumbar-sacral region in 7 (15.9%) and thoracic-lumbar-sacral region in 1 (2.3%). The median length of the interval between symptom onset and tumor diagnosis varied widely from 0 to 360 days in the entire population, however this interval was significantly shorter in patients with known neoplasia in comparisonto patients with new diagnosis (at relapse 7 days [interquartile range 3–10] vs at diagnosis 23 days [7–60]). Pre and post-operative spine magnetic resonance imagingwas performed in all cases, and most(95%) patients underwent neurosurgical treatment as first treatment. Severe motor deficit was associated with younger age and severe motor deficit at diagnosis was associated withworst motor outcomes at discharge from neurosurgery. Patients with progression or relapsed disease showed a worst prognosis, while the majority of patients (70.5%) were alive at 5 years after diagnosis.

**Conclusions:**

The natural history of MSCC in children is associated to permanent paralysis, sensory loss, and sphincter dysfunction, thus prompt diagnosis and correct management are needed to minimize morbidity. Treatment strategies differed widely among cancer types and study groups in the absence of optimal evidence-based treatment guidelines. When the diagnosis is uncertain, surgery provides an opportunity to biopsy the lesion in addition to treating the mass.

## Introduction

Malignant spinal cord compression (MSCC) is one of the most feared complications of pediatric spinal cancers. [1] Cord compression may be the presenting feature of a new malignancy, or a consequence of metastatic disease progression or relapse.[2—9]Spinal cord compression (SCC)can be classified into three groups, based on tumor location:extradural (E-SCC), intradural/extramedullary (I/E-SCC), and intramedullary (I-SCC), and can be caused by a number of ethiologicmechanisms including direct spread of tumor, extension of tumor through vertebral foramina into epidural space and bony disease within vertebrae with secondary cord compression. In adults, extradural tumors are most common, as they reside in the vertebrae body or structures outside the dura. Intradural-extramedullary tumors are the second most common and come from the leptomeninges or nerve roots inside the dura, but external from the spinal cord. The least common are intramedullary spinal cord tumors which arise from the spinal cord proper, leading to invasion and destruction of the gray and white matter [1]. Despite their impact on patient morbidity and mortality, very limited data are available on incidence and etiology of MSCC in pediatric population [2–9]. Moreover, diagnosis of MSCC in children can be particularly difficult at an early phase, especially in infants, thus increasing short- and long-term morbidity [10]. Yet, further insights in MSCC are key, as its natural history, if untreated, typically entails paralysis, sensory loss, and sphincter dysfunction, and this applies to children as well as to adults.The objective of this study was to examine etiology, clinical presentation and treatment of pediatric patient with MSCC admitted to the Santobono-Pausilipon Children’s Hospital, Naples, Italy.

## Methods

This retrospective study was carried out at the Department of Pediatric Neurosurgery and Pediatric Oncology of Santobono-Pausilipon Children’s Hospital, Naples, Italy, from January 2007 to January 2019. A neurosurgerical and oncology database of all children (age < =18 years) with solid tumors has been maintained since 2007. Children, who had documented MSCC but without related symptoms, were not considered for this study. Once children were identified from the database, their health care records were reviewed. Case definitions for MSCC were based on the etiological classifications within the International Spinal Cord Society (ISCoS) core data set for non-traumatic Spinal Cord Injury dataset [11].

### Grading of MSCC

The degree of motor deficit was evaluated by prospectively applying the Spinal Injury Association Impairment Scale adapted to patients’ age [12]. It was graded as follows: grade 1, mild hypostenia with walking disability for legs, or difficulty in raising hands above head for arms; grade 2, moderate hypostenia with inability to walk and make movements against gravity or raise the hands above the head; grade 3, severe hypostenia with paraplegia, no elicitable tendon reflexes or muscular movements. The other main presenting symptoms (sphincter dysfunctions, pain and respiratory distress) were reported as either being present or absent.

### Statistics

Descriptive statistics were used in terms of absolute frequencies and percentages for categorical variables and the Pearson’s chi-square test or Fisher’s exact test, if appropriate, was applied to compare proportions. Quantitative data were described in terms of median values with their interquartile range (IQR) and differences between groups were assessed by the Mann–Whitney Test. More advanced statistical analysis was not carried out, because the numbers in each diagnostic and etiological group were so low. All tests were two-tailed and a *P*-value < 0.05 was considered statistically significant. All data were performed by using MedCalc for Windows, version 9.6.4.0 (MedCalc Software, Mariakerke, Belgium).

## Results

### Etiology

**Fig. 1:**
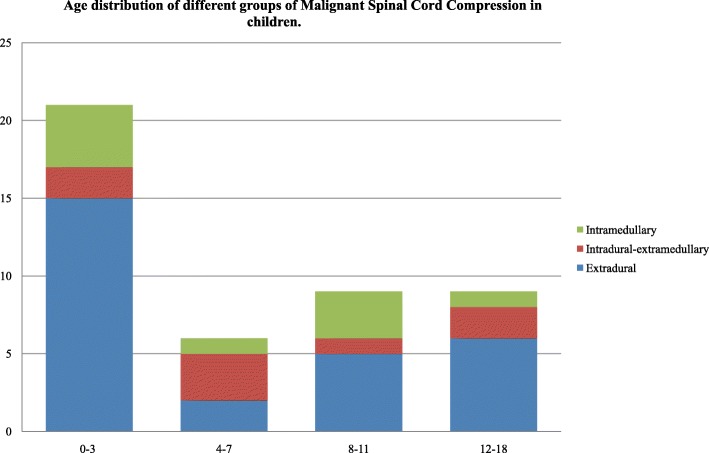
Age distribution of different groups of Malignant Spinal Cord Compression in children

Since 2007, a total of 57 patients aged 0–18 years have sustained a tumor SCC. Table 1 displays etiology of the compression. Of these 57 patients, 13 (22.8%) were excluded from analysis because affected by non-malignant tumours (NM), leaving 44 evaluable (20 male, 45.4%) including 28 (63.6%) cases of extradural, 7 (15.9%) cases of intradural/extramedullary and 9 (20.4) cases of intramedullary tumors. The median age at time of MSCC diagnosis was 52 months (interquartile [IQR] 20.5–112, range 0–205). Age distribution of patients showed a peak in young patients (≤3 years old) (Fig. 1).

**Table 1:** Etiology of Symptomatic Spinal Cord Compression in pediatric patients

*Condition*	*NM-SCC*	*M-SCC*
No of cases	13	44
Males, n (%)	6 (46.1)	20 (45.4)
Age at diagnosis of SCC, months, median (IQR; range)	95 (23–125; 4–179)	52 (20.5–112; 0–205)
Etiology, n (%)	*Extradural11 (84.5)*	*Extradural 28 (63.6)*
	Lipoma 6 (46.1)	Neuroblastoma 12 (27.2)
	Aneurysmal bone cyst 4 (30.7)	Ewing Sarcoma 7 (15.9)
	Osteoblastoma 1 (7.7)	Yolk SacTumor 2 (4.5)
	*Intradural extramedullary 2 (15.4)*	Metastatic tumors 7 (15.9)
	Meningioma 1 (7.7)	- Ewing Sarcoma (chest wall)
	Plexiform Neurofibromas 1 (7.7)	- Ewing Sarcoma (pelvis)
		- Ewing Sarcoma (spine)
		- Hepatoblastoma
		- Osteosarcoma (distalfemur)
		- Rhabdomyosarcoma (thigh)
		- Rhabdoid Tumor (kidney)
		*Intradural extramedullary 7 (15.9)*
		Malignant Schwannoma 1 (2.3)
		AT-RT 1 (2.3)
		Metastatic tumors 5 (11.3)
		- AT-RT
		- Choroid Plexus Carcinoma
		- Medulloblastoma (2)
		- Suprasellar Germ Cell Tumor
		*Intramedullary 9 (20.4)*
		Ependymoma 2 (4.5)
		Pylocitic Astrocytoma 3 (6.8)
		High-Grade Glioma 1 (2.3)
		Low-Grade Glioma 3 (6.8)

*NM* non malignant, *M* malignant, *SCC* spinal cord compression, *AT-RT* Atypical TeratoidRhabdoidTumor

### Clinical presentation

MSCC characteristics at diagnosis are provided in Table 2. Cord compression was the presenting feature of a new malignancy in 33 (75%) patients, a consequence of metastatic disease progression or relapse in the remaining 11 (25%) patients. The median length of the interval symptom-tumor diagnosis was 11 days (IQR 7–60; range 0–360) in the entire population and was between 0 and 7 days in 19 patients, 8–30 days in 11, and more than 30 days in 14. However in patients with known neoplasia the interval was significantly shorter (at relapse 7 days, IQR 2.8–10.3 vs at diagnosis 22.5 days, IQR 7–60 days, p 0.03752). Motor deficit occurred in all patients (n 44/44), and was of grade 1 in 10 (35.3%), grade 2 in 19 (43.2%), and grade 3 in 15 (34.1%). It involved the upper extremities in 5 patients, the lower extremities in 38, both extremities in 2. The occurrence of severe motor deficit was more frequent in infants (age ≤ 2 years) compared to children older than 2 years (n 10/15, 22.7% vsn 5/29, 11.4%, p 0.0027) . The frequency of the other symptoms was 59.1% (n 26/44) for pain, 43.2% (n 19/44) for sphincter dysfunction and 6.8% (n 3/44) for irritability. Pain involved upper extremities in 3 (11.5%) patients, lower extremities in 12 patients (46.1%) and back in 11 (42.3) patients. Acute respiratory distress resulting from diaphragmatic paralysis and requiring intubation and mechanical ventilation was observed in 2 patients (4.5%). Cord compression was documented by magnetic resonance imaging (MRI) in all patients (n 44/44). The primary tumor site was located in the neck in 3 (6.8%) patients, thorax in 16 (36.4%), cervico-thoracic region in 3 (6.8%), thoraco-lumbar region in 8 (18.2%), abdomen in 5 (11.4%), lumbar-sacral region in 7 (15.9%) and thoracic-lumbar-sacral region in 1 (2.3%). All patients (n 44/44) underwent spine MRI at SCC diagnosis. First line treatment was neurosurgery in 42 (95.4%) patients and chemotherapy in 2 patients (1 neuroblastoma and 1 Malignant Germ Cell Tumor) not operated for high intra-operative risk.

**Table 2:** Features of Malignant Spinal Cord Compression and Patient Characteristics at Diagnosis

*Feature/characteristic*	*At diagnosis*	
	*N*	*%*
*No of cases*	44	100
*Timing of SCC*		
At diagnosis	33	75.0
At relapse	11	25.0
*Symptom–diagnosis interval, days*		
≤ 7	19	43.2
8–30	11	25
> 30	14	31.8
*Symptoms*		
Motor deficit	44	100
Grade 1	10	22.7
Grade 2	19	43.2
Grade 3	15	34.1
Pain	26	59.1
Upper extremities	3	11.5
Lower extremities	12	46.1
Spinal	11	42.3
Irritability	3	6.8
Sphincter dysfunction	19	43.2
Respiratory distress	2	4.5
*Spinalcord MRI, N (%)*	44	100
*Level of spinal cord compression*		
Cervical	3	6.8
Cervico-thoracic	3	6.8
Thoracic	16	36.4
Thoraco-lumbar	8	18.2
Lumbar	5	11.4
Lumbar-sacral	7	15.9
Thoracic-lumbar-sacral	1	2.3
*Neurosurgical treatment*	42	95.4

### Neurosurgical treatment

**Fig. 2:**
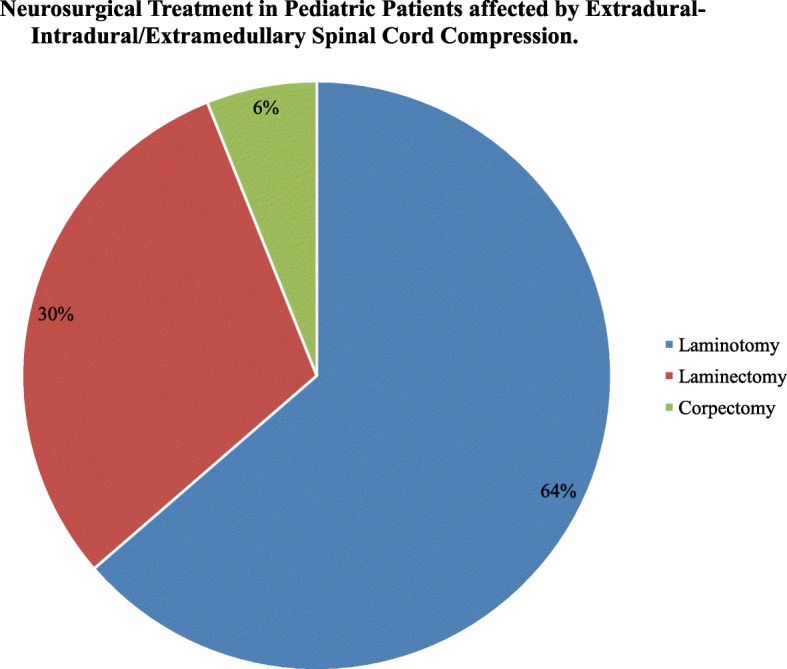
Neurosurgical Treatment in Pediatric Patients affected by Extradural-Intradural/Extramedullary Spinal Cord Compression

Main features of neurosurgical treatment of patients affected by MSCC are showed in Table 3. A total of 33 (75%) patients have sustained an E-I/E-SCC and 9 (20.4%) patients an I-SCC. The median age at time of E-I/E-SCCdiagnosis was 63 months (IQR 21–129, range 0–205) and at time of I-SCC diagnosis was 48 months (IQR 25–106, range10–198) (p ns). The length of stay in neurosurgery (6 days, IQR 3–9.3, range 2–41 for E-I/E-SCCpatients and 18 days, IQR14–20, range 10–32 for I-SCC; p 0.00112) and the interval from admission in neurosurgery and operation (E-I/E-SCCSCC0.5 days, IQR 0–1, range 0–29 versus I-SCC 7 days, IQR 3–9, range 0–18; p 0.00228) was significantly different in the two groups. The length of stay in Intensive Care Unit (ICU) was 17 h, IQR 0–24, range 0–984 for patients affected E-I/E-SCC and 24 h, IQR 0–24, range 0–50 (p ns). The majority of patients received partial removal (E-I/E-SCC26/33 vs I-SCC 7/9, p ns). There were no perioperative deaths in the two groups. Post-operative MRI scanning established adequate decompression in all patients, while reoperation was required in 1 patient for spinal stenosis within 36 h. Twenty-one (64%) patients affected with E-I/E-SCCunderwent laminotomy, 10 (30%) laminectomy and 2 (6%) corpectomy (Fig. 2).

**Table 3:** Characteristic of Neurosurgical Treatment of Patients affected by Malignant Extradural-Intradural/Extramedullary and IntramedullarySpinal Cord Compression

*Feature/characteristic*	*E-I/E*	*I*	*p*
No of cases, N (%)	33 (75)	9 (20.4)	
Age at diagnosis of SCC,months, median (IQR; range)	63 (21–129; 0–205)	48 (25–106; 10–198)	ns
Pre-operative spinalcord MRI, N (%)	33 (100)	9 (100)	ns
NS recovery, days, median (IQR; range)	6 (3–9.3; 2–41)	18 (14–20; 10–32)	0.00112
ICU recovery, hours, median (IQR; range)	17 (0–24; 0–984)	24 (0–24; 0–50)	ns
Interval NS admission-surgery, days, median (IQR; range)	0.5 (0–1; 0–29)	7 (3–9; 0–18)	0.00228
Post-operative spinal cord MRI, N (%)*	33 (100)	9 (100)	ns
NS perioperative deaths, N (%)	0 (0)	0	ns
NS perioperative complications, N (%)	4 (12.1)	0	ns
-Increased motor deficit	1 (3.0)		
-Spinal stenosis	1 (3.0)		
-Bilateral lower-limb oedema	1 (3.0)		
-Seizures	1 (3.0)		
NS resection, N (%)			ns
-biopsy	4 (12.1)	0 (0)	
-partial	26 (78.8)	7 (77.8)	
-complete	3 (9.1)	2 (22.2)	

*NS* NeuroSurgical, *ICU* Intensive Care Unit, *E* Extradural, *I/E* Intradural Extramedullary, *I* Intramedullary

*MRI within 24 h

### *Motor deficit at neurosurgery discharge in* E-I/E-SCC

Following neurosurgical treatment for E-I/E-SCC, 5 patients (11.9%) achieved complete motor recovery, 18 improved (42.8%), and 10 remained stable (23.8%) (Table 4). Complete recovery occurred in 3 of 7 patients (42.8%) with grade 1, 1 of 14 (7.1%) of those with grade 2 and 1 of 12 (8.3%) of those with grade 3 motor deficit. Seven patients (58.3%) with grade 3 and 3 patients with grade 2 motor deficit showed no improvement after surgery (Table 4).

**Table 4:** Clinical Response to Neurosurgical Treatment at discharge from Neurosurgery in Patient affected by Malignant Extradural-Intradural/ExtramedullarySpinal Cord Compression

	*No change*		*Improvement*		*Normal*	
	*N*	*%*	*N*	*%*	*N*	*%*
*Grade of Motor Deficit*						
Grade 1 (*n* = 7)	0	0	4	57.1	3	42.8
Grade 2 (*n* = 14)	3	21.4	10	71.4	1	7.1
Grade 3 (*n* = 12)	7	58.3	4	33.3	1	8.3

### Five-year survival

Thirteen patients (29.5%) died, of which 5 (38.4%) as a direct result of the tumour, 8 (61.6%) for metastatic progression or relapse. Details on individual patients are reported in Table 5

**Table 5:** Details and Long-Term Follow-up of Pediatric Patients affected Malignant Spinal Cord Compression

*ID*	*Sex*	*Level of SCC*	*Type*	*Age at SCC (months)*	*Interval symptoms -SCC diagnosis (days)*	*Motor deficit (grade)*	*Diagnosis*	*NCH*	*Status*	*Follow-up (months)*
1	F	L1-L3	E	101	7	2	Metastasis of Rhabdomyosarcoma	Yes	Dead	-
2	F	D5-D6	E	108	4	1	Ewing Sarcoma	Yes	Alive	61
3	M	D9-D11	E	195	2	2	Metastasis of Ewing Sarcoma	Yes	Alive	4
4	M	C2-D4	I	25	30	2	PylociticAstrocytoma	Yes	Alive	92
5	M	L1-L3	E	46	4	2	Neuroblastoma	Yes	Alive	8
6	M	D12-L4	E	3	2	3	Neuroblastoma	Yes	Alive	98
7	F	D4-D11	I/E	63	120	1	Metastasis of Medulloblastoma	Yes	Alive	86
8	F	D9-L1	I/E	36	7	2	Metastasis of AT-RT	Yes	Dead	-
9	M	C4-D5	E	13	7	2	Neuroblastoma	Yes	Dead	104
10	F	D6-D10	I	102	14	3	Anaplastic Ependymoma	Yes	Alive	90
11	F	L5-S1	I/E	165	60	1	Malignant Schwannoma	Yes	Alive	13
12	F	L1-S1	E	24	12	3	Neuroblastoma	Yes	Alive	77
13	M	D10-D12	E	163	60	3	Metastasis of Ewing Sarcoma	Yes	Alive	65
14	F	D10-D11	E	87	60	3	Ewing Sarcoma	Yes	Dead	-
15	F	D11-L1	E	10	7	1	Neuroblastoma	Yes	Alive	106
16	F	D7-D9	E	39	10	1	Neuroblastoma	Yes	LF	-
17	M	L5-S2	E	204	30	1	Ewing Sarcoma	Yes	Alive	24
18	F	D1-D5	E	21	7	3	Ewing Sarcoma	Yes	Dead	-
19	F	L4-S1	E	171	7	2	Metastasis of Osteosarcoma	Yes	LF	-
20	M	C2-C6	I	106	360	3	Low Grade Glioma	Yes	Alive	126
21	M	D11-S5	E	20	60	3	Malignant Germ Cell Tumor	Yes	Alive	82
22	F	D1-D3	I	42	30	2	Low Grade Glioma	Yes	Alive	28
23	F	D5-D11	I	10	90	3	PylociticAstrocytoma	Yes	Alive	104
24	F	D10	I/E	129	20	3	Metastasis of Malignant Suprasellar Germ Cell Tumor	Yes	Dead	-
25	M	C2-D2	I	48	60	2	PylociticAstrocytoma	Yes	Alive	114
26	F	D4-D10	E	41	60	1	Neuroblastoma	Yes	LF	-
27	F	D12-L2	E	134	7	2	Ewing Sarcoma	Yes	Alive	60
28	M	D8	I/E	36	2	2	Metastasis of Medulloblastoma	Yes	Dead	-
29	M	L2-S1	I/E	52	10	1	AT-RT	Yes	Dead	-
30	M	D2-D3	E	116	360	1	Ewing Sarcoma	Yes	Alive	2
31	F	L2-L4	I	107	240	1	Ependymoma	Yes	Alive	39
32	M	L2-L4	E	2	0	3	Metastasis of renal RT	Yes	Dead	-
33	M	D11-L3	E	0	0	3	Neuroblastoma	Yes	Alive	23
34	F	D8-L5	I/E	79	7	2	Metastasis of Choroid Plexus Carcinoma	Yes	Dead	-
35	M	D11-L3	E	8	2	3	Neuroblastoma	Yes	Alive	46
36	F	L2-S2	E	19	60	3	Neuroblastoma	None	Alive	60
37	M	L3-S1	I/E	147	15	2	Neuroblastoma	Yes	Dead	-
38	M	D2-D4	E	205	7	2	Metastasis of Ewing Sarcoma	Yes	Dead	-
39	M	C5	E	90	3	2	Metastasis of Hepatoblastoma	Yes	Dead	-
40	F	L5	I	198	30	2	High Grade Glioma	Yes	Alive	2
41	F	D10-L2	I	18	180	2	Ganglioglioma	Yes	Alive	94
42	F	D7-D9, L3	E	14	60	2	Malignant Germ Cell Tumor (Yolk Sac)	None	Alive	104
43	M	D2-D6	E	11	9	3	Neuroblastoma	Yes	Alive	91
44	F	C6	E	101	7	2	Ewing Sarcoma	Yes	Alive	42

*LF* Lost to Follow-up, *AT-RT* Atypical Teratoid-Rhaboid Tumor, *RT* Rhaboid Tumor

## Discussion

Spinal cord compression secondary to cancer is a rare diagnosis but represents an oncology emergency as itmay lead quickly to permanent paralysis, if not treated effectively and promptly. Acute compression of the spinal cord occurs in 3 to 5% of children with cancer, often at diagnosis [13, 14]. To assess etiology, clinical presentation and treatment of MSCC we evaluated retrospectively 44 children under 18 years with symptomatic MSCC. The median age of our patients was 52 months while Tantawy et al. reported that 8 years was the mean age of their patients [7]. During the study period the leading cause of MSCC in children under 18 years was extramedullary tumors (63.6%), in particular neuroblastoma (27.2%) followed by Ewing sarcomas (15.9%), similarly to previous studies [7–9]. Cord compression was the presenting symptoms of a new cancer in 75% of cases.Motor deficit was the initial symptoms of spinal compression in all patients, while pain was present in about 60% of patients, followed by sphincteric deficit (43.2%). Almost the same observation was reported by De Bernardi et al., who reported that motor deficit was the most presenting MSCC symptom (98.7%) followed by pain (61.8%) and then sphincteric dysfunction (39.5%) [6, 15]. The median length of the interval symptom-tumor diagnosis varied widely from 0 to 360 days in the entire population, however this interval was significantly shorter in patients with known neoplasia respect to patients with new diagnosis (at relapse 7 days, IQR 2.8–10.3 vs at diagnosis 22.5 days, IQR 7–60 days). It is widely accepted that MSCC is considered as a medical emergency and any diagnostic delay should be avoided. For children presenting in District General Hospitals with a strong suspicion of SCC, transfer to a principal treatment centreis recommended because management is highly complex and requires the input of a Neurosurgery and Clinical Oncology. In our case series, pre and post-operative spine MRI was performed in all the cases It is widely recognized that pre and post contrast MRI spine is the gold standard for diagnosis, and should be performed before 24 h have elapsed [16]. In our study, severe motor deficit was associated with younger age and severe motor deficit at diagnosis was associated to worst motor outcomes at discharge from neurosurgery. Our patients with progression or relapsed disease showed a worst prognosis, while the majority of patients (70.5%) were alive at 5 years after diagnosis. Recently, a systematic review showed that patients with SCC due to NBL differ from patients without intraspinal extension with younger age at diagnosis. Moreover the severity of the neurological motor deficit at diagnosis had the most predictive power for the neurological outcome [17].

Classification in E, I/E and I-SCC is helpful in developing a differential diagnosis and guiding appropriate clinical management. Surgical resection is the treatment of choice for I-spinal tumors leading to SCC. Instead, the discussion whether to perform immediate surgical decompression in cases of E-I/E spinal cord compromise due to cancer is still open. In our case series, the majority of patients (~ 95%) underwent neurosurgical treatment as first treatment with adequate decompression, independently of the etiology and there were no perioperative deaths. Children with E and I/E underwent neurosurgical treatment in less than 24 h, while patients with I-tumors in about 7 days. Due to the complexity of the surgery in case of I-SCC, patients stayed longer in ICU and neurosurgical department.In case of E-I/E-SCC the primary role of neurosurgery has been replaced for chemotherapy since Hayes et al. start pioneering the use of primary chemotherapy in management of NBL with intraspinal extension [18]. Results of various retrospective studies showed that chemotherapy can be an effective initial treatment option in NBL and Ewing Sarcoma,however population size of these studies is too small to make definite conclusions. [15, 17, 19]. Actually, a SIOPEN prospective study registry of Peripheral NeuroblasticTumours (PNTs) presenting with spinal canal involvement aims to describe the natural history of PNTs presenting with SCC and describe the diagnostic and therapeutic approaches adopted in participating centers. As results from this and other clinical studies will become available, physicians will be able to make better-informed decisions on treatment for future pediatric patients.

Limitations of this work are several, and of course include the retrospective, observational, single-center design. In addition, we did not focus on the inclusion of asymptomatic MSCC, and thus additional studies are required to confirm our findings in general, and in particular to detail the outlook of MSCC without symptoms or clinical signs.

## **Conclusions**

The natural history of MSCC typically entails a poor prognosis as well as permanent paralysis, sensory loss, and sphincter dysfunction. This case series emphasize the need to consider cancer in the differential diagnosis of more common diseases in pediatric patients, especially because early diagnosis and proactive treatment are vital to improve prognosis and survival. For children with a high suspicion of MSCC, transfer to a principal centreis recommended because management is complex and requires multidisciplinary discussion about best treatment. Moreover, a child with history of cancer who develops back/extremity pain should be considered to have spinal cord compromise until proved otherwise. Spine MRI is the gold standard for diagnosis, and should be performed before 24 h have elapsed. Treatment strategies differ widely among cancer types and study groups in the absence of optimal evidence-based treatment guidelines. When the diagnosis is uncertain, surgery provides an opportunity to biopsy the lesion in addition to treating the mass.

## Data Availability

The dataset and analyses are available from the corresponding author on reasonable request.
